# Effects of stroboscopic visual training on reaction time and decision-making ability in athletes: a systematic review and meta-analysis

**DOI:** 10.3389/fpsyg.2025.1697425

**Published:** 2025-11-17

**Authors:** Rishu Wang, Sheng Li, Yidong Wu, Hengxian Liu, Qi Zhang

**Affiliations:** 1 School of Athletic Performance, Shanghai University of Sport, Shanghai, China; 2 School of Wulinfeng Sports, Haikou University of Economics, Haikou, China; 3 School of Economics and Management, Shanghai University of Sport, Shanghai, China

**Keywords:** stroboscopic visual training, reaction time, decision-making ability, athletes, systematic review, meta-analysis

## Abstract

**Introduction:**

This systematic review and meta-analysis investigated the impact of stroboscopic vision training on athletes’ reaction and decision-making ability, and examined the moderating role of key factors.

**Methods:**

Literature searches were conducted in five databases: PubMed, Web of Science, Embase, EBSCO and Scopus. Two researchers independently screened the articles and extracted the data. The risk of bias in the included studies was evaluated using the Cochrane risk of bias assessment tool. Statistical analysis was conducted using Stata 18.0 software. Meta-analysis employed a random-effects model to analyze reaction time and decision-making ability, respectively. Subgroup analysis studied the moderating effects of participants’ age, sport experience, sport type, stroboscopic frequency, duty cycle, total intervention duration, weeks of intervention, intervention frequency and per session intervention duration. A total of 9 articles were included in the systematic review and meta-analysis, involving 323 athletes.

**Results:**

Studies have shown that stroboscopic vision training has a significant impact on the improvement of reaction time (SMD = −0.82, 95% CI: −1.42, −0.22, *p* = 0.007), but has no significant effect on decision-making ability (SMD = 0.51, 95% CI: −0.09, −1.11, *p* = 0.09). Subgroup analysis indicates that stroboscopic training for 1 to 6 weeks, with 1 to 2 sessions per week and 10 min of high-quality training each time, is an excellent training program for optimizing athletes’ reaction ability. Duty cycles of less than 10 Hz and less than 50% are more beneficial for improving athletes’ reaction ability (SMD = −1.38, *p* < 0.05; SMD = −1.38, *p* < 0.05). Strobe training has different effects on different sports types, and the performance of open skill athletes (SMD = −0.60, *p* < 0.05) was significantly better than that of closed skill athletes (SMD = −2.02, *p* > 0.05). The cognitive performance of adolescent athletes under the age of 18 improved to a certain extent after stroboscopic training (SMD = −0.32, *p* = 0.05). The study suggests that stroboscope training has little impact on decision-making ability. Only training experience (≥7.5 years: SMD = −3.9, *p* < 0.001) and short training time (≤10 min: SMD = −3.9, *p* < 0.001) have positive effects on decision-making ability.

**Discussion:**

Based on this, these findings provide certain evidence for researchers and coaches, which can assist them in conducting stroboscopic training. However, due to the limitation of a small research sample size, further studies on optimizing stroboscopic training schemes are needed in the future to maximize the cognitive performance of athletes.

**Systematic review registration:**

CRD42023418594, https://www.prisma-statement.org/prisma-2020.

## 1 Introduction

In high-level sports competitions, the outcome of a game often depends on the slightest detail, forcing athletes to face increasing pressure when processing visual information. If athletes cannot accurately and quickly perceive and predict their opponents’ behaviors, they are likely to lose the best offensive or defensive opportunities (Burhaeın and Sumantri, 2024). In team sports (such as basketball and football), the accuracy of shooting or passing is closely related to reaction speed (Jin et al., 2023). In skill-dominant sports (such as tennis and badminton), high serve velocities place even greater demands on an athlete’s reaction ability, where a delay of even 20 milliseconds in reaction time can be the difference between a successful return and an error (Lochhead et al., 2024). In other words, cognitive ability (such as visual response ability) is a key factor in determining competition results. Consequently, the pursuit of effective training methods to enhance athletes’ visual-cognitive performance has emerged as a vital line of research for both scientists and coaching teams in the field of sports science.

Nowadays, the attention paid to stroboscopic vision training (SVT) has increased significantly, and it has now become a popular tool for motor vision training. The basic theory of SVT is to provide progressive stimulation to the visual motor system by adjusting the frequency and duty cycle of stroboscopic glasses. SVT mainly employs specialized stroboscopic glasses (also known as shutter glasses, such as Nike Strobe or Senaptec Strobe), and the training is usually completed by alternating between transparent and opaque lens states. Athletes enhance their visual processing and perceptual cognitive abilities in real competition scenarios by wearing stroboscopic glasses to complete specific sports tasks. This training method intentionally increases the difficulty of visual training to induce adaptive responses (Hülsdünker et al., 2021a). This adaptive response can efficiently complete visual motion processing under standard visual conditions and be transformed into excellent motor performance.

In the field of sports science, SVT has gained significant recognition and is gradually being applied to the career development plans of elite athletes. The benefits of SVT extend to an athlete’s career trajectory by building a more resilient and efficient cognitive system. This fosters the sustained high performance and adaptability required for a long and successful career at the elite level. Researchers have found that SVT can improve central visual field sensitivity, short-term memory and predictive decision-making (Appelbaum et al., 2011, 2012; Smith and Mitroff, 2012; Mitroff et al., 2013). Subsequent studies also explored the impact of SVT on aspects such as reaction speed, visual agility, motion perception, visual attention, capture performance, visuomotor performance and anticipation skill (Wilkins and Gray, 2015; Wilkins et al., 2018; HülSdünker et al., 2019; Zwierko et al., 2022; Zwierko T, et al., 2024). SVT holds that it can enhance reaction ability, predictive ability, visual memory and motor coordination under partially occluded visual conditions. Visual reaction time refers to the ability to perform rapid and precise movements of the body or a part of the body in response to a visual stimulus. It encompasses both the reaction time—from the onset of the stimulus to the initiation of movement—and the time required for the execution of the movement (Palmer et al., 2022). In sports competitions, athletes usually respond to visual stimuli based on agility (Hülsdünker et al., 2021b). Six weeks of stroboscopic vision training significantly enhanced the visual response ability (reaction time, reaction speed and agility) of volleyball players (Strainchamps et al., 2023). Then, it was shown that a six-week SVT intervention significantly improved the hitting accuracy of the stroboscopic group of tennis players (Zwierko et al., 2024b). In addition, Researchers have further confirmed these positive effects in electroencephalogram studies. For instance, neurophysiological evidence indicates that SVT enhances neural communication through the dorsal visual stream, largely mediated by magnocellular pathways. This training method induces phase rest of alpha oscillations within the occipital cortex and facilitates cross-frequency coupling between alpha and gamma rhythms, in addition to boosting predictive coding mechanisms in the parietal cortex (Ballester et al., 2017). EEG findings further demonstrate that a 4–6 week SVT intervention significantly reduces P100 latency and elevates the amplitude of visual evoked potentials reflecting both faster and enhanced early visual processing of dynamic information (Zwierko et al., 2023). Therefore, stroboscopic vision training can enhance reaction ability or reaction speed through a multi-sensory weighting mechanism.

However, the influence of stroboscopic training on different indicators in the field of perception and cognition varies. For instance, after international table tennis players wore stroboscopic glasses for 10 min of specialized warm-up exercises, their reaction time was significantly reduced, but there was no significant change in their hitting time (Schootemeijer and Visch, 2017). Stroboscopic training cannot significantly enhance the visual search ability of volleyball players (Palmer et al., 2022). These pieces of evidence may suggest that stroboscopic training is not applicable to all perceptual and cognitive abilities. In the research on how stroboscopic training affects athletes’ decision-making, it was found that the ability of football players to predict the accuracy of passing and shooting directions when completing video decision-making tasks by wearing stroboscopic glasses was significantly improved (Zwierko et al., 2024b). However a large number of research evidence indicates that SVT can lead to near transfer of perceptual cognitive functions (reaction time, agility, short-term memory, anticipatory decision-making, etc.), but the evidence for far transfer of sport performance is still uncertain (Holliday, 2013). A transfer of skills is the generalization of skills that are acquired through training across different domains (Fransen, 2024). Near transfer occurs when the training situation and behavior are similar to those in real competitions. Conversely, distant migration will occur (Sala et al., 2019). For instance, basketball dribbling skills training can make him an excellent ball handler, but it may not affect his physical fitness. We believe if there are differences among the intervention plans (such as the frequency of stroboscopic glasses, intervention time, intervention methods, etc.), the subject groups (such as team sports, individual sports, etc.), and the outcome variables (such as reaction time, the number of gazes, gaze time, hand-eye coordination ability, accuracy of predictive decision-making, etc.). Then, the overall effectiveness of SVT on athletes’ perceptual cognitive abilities and sport performance may remain undetermined, which would limit the comparison among different results (Luo et al., 2025).

Nowadays, research on athletes’ cognitive and decision-making abilities using SVT is gradually increasing. However, the meta-analysis aspect in this field has not yet been deeply explored, and systematic and quantitative evaluations are still lacking. As of now, there is insufficient evidence regarding how SVT affects motor performance and its effects, nor has a clear result been provided. This further highlights the urgency of conducting a comprehensive, integrated and systematic assessment of this field, thereby elaborating on the extent to which SVT affects athletes’ various sports performances and exploring the potential moderating factors that cause differences in the impact effects. In conclusion, this study systematically retrieved the literature on the impact of SVT intervention on athletes’ response ability and decision-making ability, and explored the potential regulatory mechanisms. Based on the research review, SVT is most commonly applied in sports that enhance reaction time and decision-making ability. Therefore, this study employs systematic review and meta-analysis methods to comprehensively evaluate the effectiveness of SVT in improving athletes’ reaction time and decision-making ability. In addition, the research will also explore the moderating effects of key factors (such as sports experience, total intervention duration, intervention frequency, and stroboscopic frequency, etc.) on training outcomes. This study aims to provide evidence for SVT in improving athletes’ reaction time and decision-making ability, and to offer a theoretical basis and practical guidance for the development of SVT programs based on enhancing sports performance.

## 2 Methods

This study was conducted in compliance with Preferred Reporting Items for Systematic Reviews and Meta-Analyses (PRISMA) 2020 and was registered at International Prospective Register of Systematic Reviews (PROSPERO), under number (CRD42023418594).

### 2.1 Search strategies

We conducted a systematic and comprehensive search in five English databases: PubMed, Web of Science, Embase, EBSCO and Scopus. Two independent researchers conducted an initial screening of the literature titles and abstracts. The main search focuses on relevant articles published before July 31, 2025. In each database, the keywords used for retrieval are as follows: (Stroboscope visual training, Stroboscope training, Strobe training, Stroboscopic, Perceptual-cognitive, Visual function, Visuomotor reaction, Reaction speed, Reaction agility, Decision-making, Sports performance, Athletic performance, Athletes, Professional athletes). These terms were combined in various configurations using Boolean operators (AND, OR) and applied uniformly across all databases. As in Appendix 1.

### 2.2 Inclusion and exclusion criteria

We designed the inclusion and exclusion criteria according to the PICOS principle (Costantino et al., 2015). We imported all records from the databases into the reference management software EndNote 20. We applied the following inclusion criteria: ① Participants (P): healthy athletes (professional, semi-professional, or amateur). ② I ntervention (I): use of wearable stroboscopic devices (e.g., Senaptec Strobe, Nike Vapor Strobe, PLATO goggles). ③ Comparison (C): control groups using non-stroboscopic settings (e.g., devices without strobe function) or conventional visual training. ④ Outcomes (O): sport-specific performance outcomes, including time-based measures (reaction time, reaction speed, reactive agility) and accuracy-based measures (decision-making accuracy, score). ⑤ Study design (S): randomized controlled trials (RCTs) published in English. We excluded studies based on the following criteria: ① Participants were para-athletes, individuals undergoing injury rehabilitation, or other special populations. ② Interventions included additional forms of sensory stimulation (e.g., auditory cues, tactile feedback, virtual reality, augmented reality, or multimodal perceptual-cognitive training) that could confound the isolated effects of stroboscopic training. ③ Studies lacked a clearly defined control group, or the control intervention was not comparable to stroboscopic training. ④ Outcomes were not directly related to sport-specific performance (e.g., balance, spatial judgment, visual search ability, EEG). ⑤ Study designs were non-RCTs, qualitative studies, case reports, reviews, conference abstracts, or studies with incomplete data.

The full text of studies that met the criteria was further reviewed by two independent researchers after an initial screening based on inclusion and exclusion criteria. If two researchers disagree on the assessment of the research literature reviewed, a third researcher is consulted and a decision made. A total of 9 eligible and relevant papers were finally included.

### 2.3 Data extraction and coding

The literature search was independently conducted by two researchers. Upon completion of the initial search, preliminary results were reviewed and cross-verified by two researchers to ensure the accuracy, completeness, and consistency of the process. During the screening phase, titles and abstracts were independently assessed by two researchers. For studies requiring full-text review, any discrepancies were resolved through third-party adjudication by one researcher, who also facilitated online discussions. The entire literature identification and selection process was conducted in strict accordance with PRISMA guidelines. EndNote 20 was used for reference management to maintain standardization and reproducibility throughout all stages of the review.

The extracted content included: (1) Basic bibliographic details, such as author, country, and year of publication. (2) Participant characteristics, including sample size, age, sex, sports, years of training and sports experience. (3) Intervention details, encompassing study design, intervention duration, training frequency, type of stroboscopic device, testing instruments, and performance outcome (time-based or accuracy-based). (4) Outcome measures, specifically time-based and accuracy-based sport-specific performance indicators, reported as mean ± standard deviation. For potential moderator variables-such as intervention duration, frequency, session length, strobe frequency, participant age and experience years-mean values were extracted. (5) Studies reporting multiple time-based or accuracy-based outcomes were treated as containing multiple independent entries. (6) Strobe frequency was classified into low, medium, and high ranges according to predefined criteria. In cases of missing data, corresponding authors were contacted to obtain the necessary information. If no response was received within 48 h, a follow-up email was sent. Studies were excluded if no reply was received within an additional 48 h.

### 2.4 Quality assessment

The risk of bias in the included randomized controlled trials was systematically assessed using the second version of the Cochrane-endorsed Risk of Bias tool (RoB 2). This tool evaluates potential sources of systematic bias across seven predefined domains: (1) Random sequence generation (selection bias). (2) Allocation concealment (selection bias). (3) Blinding of participants and personnel (performance bias). (4) Blinding of outcome assessment (detection bias). (5) Incomplete outcome data (attrition bias). (6) Selective reporting (reporting bias). (7) Other bias. Assessments were based on information extracted from full-text articles, supplementary materials, trial registrations, and, where available, additional data provided by the original study authors. Two reviewers independently performed the RoB 2 assessments. If there are serious disagreements on items, they will discuss with a third researcher.

The methodological quality of randomized controlled trials was evaluated using the Physiotherapy Evidence Database (PEDro) scale. The PEDro scale consists of 11 items, among which the following 10 parts are included in the scoring system: (1) random allocation, (2) allocation concealment, (3) baseline comparability, (4) blinding of participants, (5) therapist, and (6) assessor, (7) completeness of outcome, (8) intention-to-treat (ITT) analysis, (9) between-group comparisons, and (10) reporting of outcome variability. Each dimension is scored as either 1 or 0. The higher the score, the higher the quality of the research is considered. The PEDro assessment was initially conducted independently by Wang and Wu. If there are serious differences in the assessment, they will discuss them with the third researcher.

### 2.5 Statistical analysis

This study conducted statistical analysis using Stata version 18.0 and Review Manager 5.3. Meta-analysis uses the mean and standard deviation to represent the effect size, and the 95% CI represents the estimated interval of the population parameters constructed from the sample size. Heterogeneity tests were conducted using the Q test and I^2^ statistics. If I^2^ < 50% and *p* > 0.1, it is considered that the heterogeneity of the study is relatively small, and the fixed-effect model is selected for analysis. If I^2^ > 50% and *p* < 0.1, it is considered that the heterogeneity of the study is relatively large, and the random effects model is selected for analysis (Cheung and Cheung, 2016). Due to the small sample size of the included studies, this study used Hedge’s g (Nagashima et al., 2019) to calculate the effect size. The classification of Hedge’s g effect sizes is as follows: Small (<0.2), moderate (0.2 ≤ SMD ≤ 0.5), large (0.5 ≤ SMD < 0.8), and very large (SMD ≥ 0.8; Hedges et al., 2010). In addition, in this study, funnel plots and Egger’s were used to evaluate publication bias. A *p* < 0.05 indicated no bias. If potential publication bias occurred, the trim-and-fill method was used to adjust it to potential publication bias to ensure the objectivity of the results. Sensitivity analysis was conducted using the method of elimination one by one. For statistical tests, a *p* value <0.05 is considered statistically significant.

## 3 Results

### 3.1 Literature search results

**Figure 1:**
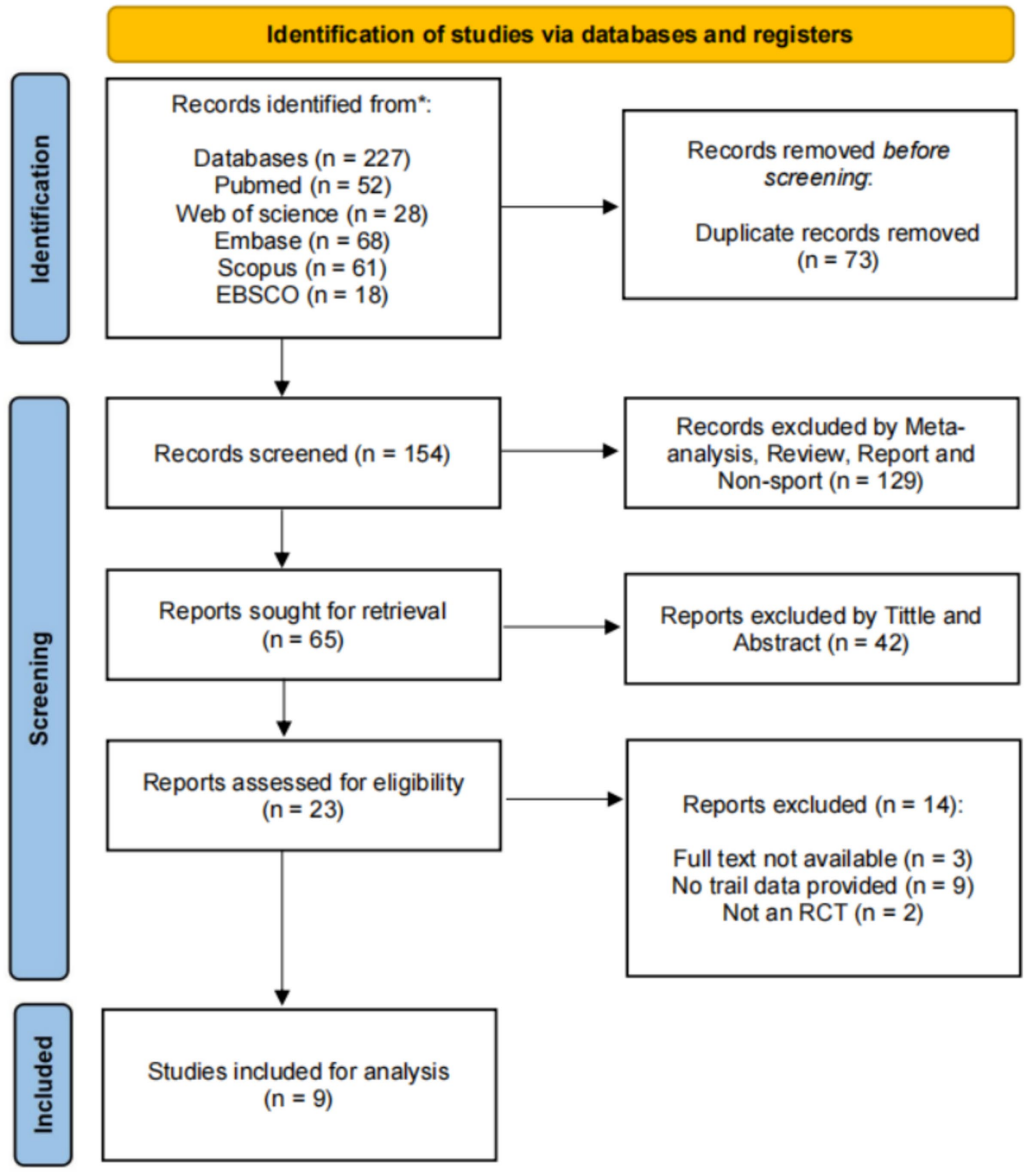
Literature screening process.

This study initially identified 227 studies through a database search. After removing 73 duplicate studies using EndNote 20, 154 studies were entered into the preliminary analysis. After being excluded by meta-analysis, review, report and non-sport (*n* = 129). 65 studies were recorded sought for retrieval. After screening for titles and abstracts, 42 studies were excluded as they did not meet the inclusion criteria. Then, 23 studies were assessed for eligibility. However, 14 studies were excluded due to full text not available, no trial data provided and not an RCT. Ultimately, 9 studies fully met the inclusion criteria of this study and were included in the final systematic review (Smith and Mitroff, 2012; Ellison et al., 2020; Sudesan et al., 2023; Zwierko et al., 2023; Gao et al., 2024; Li et al., 2024; Vasile and Stănescu, 2024; Zwierko et al., 2024b; Fortes et al., 2025). The literature screening process is shown in Figure 1. The flowchart is made from https://www.prisma-statement.org/prisma-2020-flow-diagram.

### 3.2 Basic characteristics of the included studies

This study included 9 randomized controlled trials (RCTs), which investigated the impact of stroboscopic visual training on athletes’ perceptual-cognitive abilities and athletic performance. These studies cover football, volleyball, cricket, trap shooting and curling, fully demonstrating the wide application of stroboscopic visual training in sports. The participants were mostly teenagers or athletes who had just reached adulthood, aged between 16 and 25, with an average age of 16.4 to 25.2. The study involved athletes with 3.3 to 9.96 years of sports experience. In terms of the types of sports, motor skills can be divided into open-skills and closed-skills. The former refers to the skills of performing movement tasks in an unpredictable environment, requiring individuals to respond and adjust their movements according to changes in the environment; the latter refers to the skills of performing movement tasks in a stable, predictable environment, where individuals can pre-plan their movement procedures (Zhang, 2021). There were 4 open skill sports, 3 closed skill sports, and the types of sports were not clearly defined in two studies. In the intervention trial protocol, the athletes in the experimental group need to wear stroboscopic vision glasses for training. The stroboscopic frequency is generally between 4.0 and 15.0 Hz, and the duty cycle is usually between 40 and 47.62%. The duration of the intervention is generally 1 to 8 weeks, with a total intervention duration of 7 to 784 min. The intervention frequency per week is 1 to 3 times, and each intervention session lasts for 5 to 120 min. The control group also received the same training, but they did not wear stroboscopic vision glasses. The result variables mainly include reaction time, reaction speed and reaction agility. Decision-making accuracy or score (as shown in Table 1).

**Table 1:** Basic characteristics of included studies.

Study (Years)	Participants characteristics (n/age/sport experience)	Intervention and control measure		Outcomes
		Methods	Duration	
Smith and Mitroff (2012)	SG(15/22.80 ± 2.11/N) CG(15/23.60 ± 2.82/N)	SG: Wore stroboscopic eyewear (level 3, 4 Hz, 100 ms clear, 150 ms opaque). CG: Conventional training without stroboscopic stimulation.	Single session 5-7 min	Reaction time (ms)
Ellison et al. (2020)	SG(31/20.82 ± 1.54/N) CG(31/21.34 ± 4.27/N)	SG: Wore stroboscopic eyewear (level 3, 4 Hz, 100 ms clear, 150 ms opaque). CG: Conventional training without stroboscopic stimulation.	Single session ×7-8 min	Reaction time (ms) Accuracy (%)
Sudesan et al. (2023)	SG(15/19-25/N) CG(15/19-25/N)	SG: Participants practiced each drill for a short time without the glasses first, then for a lengthy time with the glasses on, and then for a short time without the glasses to complete the Sessions (level 3, 4 Hz, 100 ms clear, 150 ms opaque). CG: Conventional training without stroboscopic stimulation.	Single session ×7-8 min	Reaction time (ms)
Zwierko et al. (2023)	SG(25/16.40 ± 0.70/6.70 ± 1.10) CG(25/16.60 ± 0.50/6.60 ± 1.30)	SG: Wore stroboscopic eyewear during volleyball-specific functional training (frequency: 15 ~ 9 Hz; duty cycle: 50% ~ 70%). Each cycle: 5 min (2.5 min rest + 2.5 min training). CG: Conventional volleyball training without stroboscopic stimulation.	6 weeks/3 sessions per week/25–30 min per session	Simple reaction time (ms) Complex reaction speed (ms) Reaction agility (ms)
Zwierko et al. (2024a)	SG(25/16.40 ± 0.70/6.70 ± 1.10) CG(25/16.60 ± 0.50/6.60 ± 1.30)	SG: Wore stroboscopic eyewear during volleyball-specific functional training (frequency: 15–9 Hz; duty cycle: 50%–70%). Each cycle: 5 min (2.5 min rest + 2.5 min training). CG: Conventional volleyball training without stroboscopic stimulation.	6 weeks /3 sessions per week/25–30 min per session	Reaction agility (ms)
Vasile and Stănescu (2024)	SG(9/16.59 ± 2.00/6.94 ± 3.01) CG(8/16.59 ± 2.00/6.94 ± 3.01)	SG: The athletes climbed with the glasses on their normal training walls: on the bouldering wall, on the lead wall, on the MoonBoard, and on the Spraywall. CG: Conventional training without stroboscopic stimulation.	20 sessions total/3–7 sessions per week/120 min per session	Simple reaction time (ms) Choice reaction time (ms) Memory access reaction time (ms)
Li et al. (2024)	SG(15/21.70 ± 1.30/3.30 ± 1.80) CG(15/21.70 ± 1.30/3.30 ± 1.80)	A ‘ladder’ drill targeting the house (effective zones 4–10), with the sequence of training increasing and then decreasing in complexity. The goal was to maintain the stone within the designated target zone before proceeding to the next one.	4 weeks 3 × 40 min	Score (points)
Gao et al. (2024)	SG(13/24.18 ± 5.12/9.96 ± 4.86) CG(13/24.18 ± 5.12/9.96 ± 4.86)	Training sessions included a variety of skills ranging from face-to-face drills, facing wall drills, and turn-and-catch exercises. In the same exercises, the control group performed the exercises under normal visual conditions while the experimental group wore Senaptec strobe glasses. Athletes went from level 1, where they did basic reaction time and tracking exercises.	8 weeks/2 sessions per week/49 min per session	Reaction time (ms) Score (points)
Fortes et al. (2025)	SG(14/25.20 ± 4.70/7.50 ± 1.70) CG(14/25.20 ± 4.70/7.50 ± 1.70)	SG: Wore stroboscopic eyewear during small-field soccer training (frequency: 4.76 Hz; duty cycle: 47.62%). CG: Wore fixed-frequency stroboscopic eyewear without active flashing.	8 weeks /3 times per week/24 min per session	Response time (ms) Accuracy (%)

### 3.3 Quality assessment of included studies

**Figure 2:**
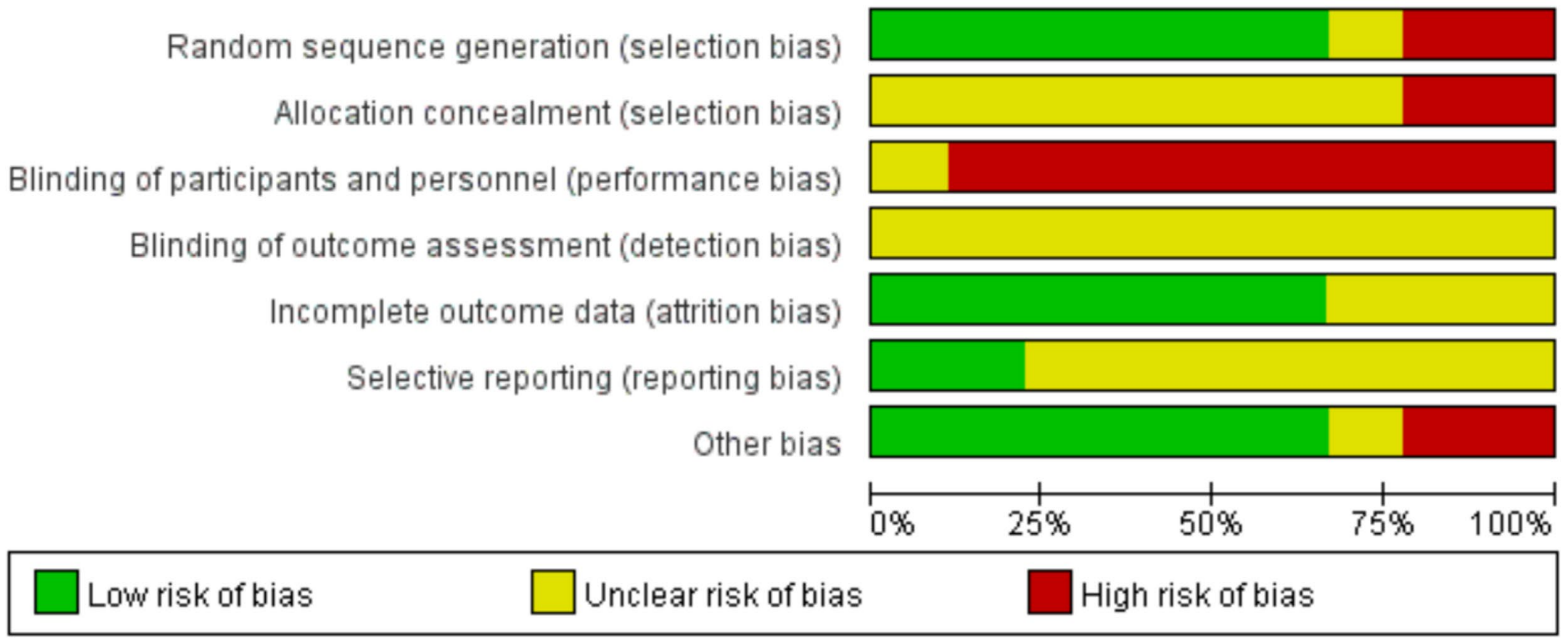
Risk of bias assessment.

In this study, the risk of bias of the included studies was evaluated using the RoB2 tool, which consists of 7 dimensions. We conducted a visual analysis through Review Manager 5.4 (as shown in Figure 2). The analysis results show that due to the special nature of the intervention measure (wearing stroboscopic vision glasses), it is impossible to blind the subjects and researchers, which may have an impact on the results. Furthermore, most studies did not elaborate on the allocation of hidden schemes (*n* = 7) and the blinding of evaluators (*n* = 9). Despite these limitations, the randomization methods of the studies were appropriate, the data integrity was good, and the risk of bias in other aspects was reduced.

It can be seen from the results of the methodological quality assessment that in most studies, the allocation concealment, implementation blinding and analysis principles were not clearly marked. Moreover, most of the studies did not blind the subjects and personnel. However, most studies performed well in terms of random allocation, two-group comparison, data and baseline information (as shown in Table 2). Overall, the included studies demonstrated moderate quality, indicating that the rigor and scientific nature of the research design were relatively reasonable. Great caution is still needed when interpreting the research results and conclusions.

**Table 2:** Methodological quality assessment results (PEDro scale).

Study (Years)	(1)	(2)	(3)	(4)	(5)	(6)	(7)	(8)	(9)	(10)	Score
Smith and Mitroff (2012)	?	?	1	0	0	?	?	?	1	1	**3**
Ellison et al. (2020)	1	?	1	0	0	?	?	?	1	1	**4**
Sudesan et al. (2023)	0	0	1	0	0	?	?	?	1	1	**3**
Zwierko et al. (2023)	1	?	1	0	0	?	?	?	1	1	**4**
Zwierko et al. (2024a)	1	?	1	0	0	?	?	?	1	1	**4**
Vasile and Stănescu (2024)	0	0	1	0	0	?	?	?	1	1	**3**
Li et al. (2024)	?	?	?	0	0	?	?	?	1	1	**2**
Gao et al. (2024)	1	?	1	0	0	?	?	?	1	1	**4**
Fortes et al. (2025)	1	?	1	0	0	?	1	?	1	1	**5**

Bold font refers to the total score of quality evaluation for all articles included in this study, which was scored from 10 dimensions respectively.

### 3.4 Pooled effect size

The results of the meta-analysis of the random effects model show that stroboscopic vision training has a significant positive effect on improving the reaction time of athletes, with significant statistical significance. The results of the seven studies combined indicated that the overall effect size was moderate to large (SMD = −0.82, 95% CI: −1.42, −0.22, *p* = 0.007). A negative effect size, in a meta-analysis of response time, typically indicates that the response time of the experimental group is shorter than that of the control group (i.e., it performs better). This indicates that compared with the control group, the reaction time of the athletes who underwent stroboscopic vision training was significantly shortened after the intervention. However, there is a high degree of heterogeneity among the studies (I^2^ = 80%), which indicates that the effect of stroboscopic training may vary significantly due to specific intervention programs or participant characteristics. To prove that stroboscopic vision training is an effective method to improve athletes’ reaction speed, it is necessary to further confirm the best training plan.

Meta-analysis of the random effects model showed that the decision-making ability of the experimental group was slightly higher than that of the control group, indicating that stroboscopic vision training had a certain positive impact on decision-making ability. However, the overall effect did not have significant statistical significance (SMD = 0.51, 95% CI: −0.09, −1.11, *p* = 0.09). Furthermore, there was moderate heterogeneity among the studies (I^2^ = 66%), indicating that the stroboscopic training effect might be influenced by moderating factors such as intervention protocols or participant characteristics.

### 3.5 Subgroup analysis

#### 3.5.1 Subgroup analysis of reaction time indicators

The results of subgroup analysis indicated that the reaction time of adolescents under 18 showed a significant improvement in marginalization (SMD = −0.32, *p* = 0.05), while adult athletes (>18) did not show statistical significance (SMD = −1.38, *p* > 0.05). For athletes with sports experience of ≥7 years (SMD = −0.35, *p* > 0.05) or <7 years (SMD = −2.02, *p* > 0.05), there was no significant difference in reaction time. In terms of sports types, the reaction time of athletes in open sports events was significantly improved, showing significant importance (SMD = −0.60, *p* < 0.05), while athletes in closed sports events did not show significant importance (SMD = −2.02, *p* > 0.05). In terms of stroboscope frequency, the training effect of < 10 Hz was significant (SMD = −1.38, *p* < 0.05), while the training effect of ≥10 Hz was not significant (SMD = −0.35, *p* > 0.05). A duty cycle of ≤50% can significantly improve the reaction time (SMD = −1.38, *p* < 0.05), while the effect of a duty cycle of >50% is not significant (SMD = −0.35, *p* > 0.05). When the total intervention duration was ≤10 min, the improvement of the response time effect showed marginal significance (SMD = −0.68, *p* = 0.05), while when it exceeded 100 min, the response time did not show significant improvement (SMD = −1.03, *p* > 0.05). The intervention period of less than 6 weeks improved the response time to a certain extent, showing a marginally significant effect (SMD = −0.68, *p* = 0.05), while the intervention period exceeding 6 weeks did not make the response time reach statistical significance (SMD = −1.03, *p* > 0.07). Training ≤2 times per week could significantly improve the reaction time (SMD = −3.90, *p* < 0.001), and only one training session could also improve the reaction time, but it showed a marginally significant effect (SMD = −0.68, *p* = 0.05), while training ≥3 times per week could not significantly improve the reaction time (SMD = −0.32, *p* > 0.05). Each training session lasting less than 10 min can improve the reaction time, but only shows marginal significance (SMD = −0.68, *p* = 0.05). However, each training session lasting ≥25 min cannot significantly improve the reaction time (SMD = −1.03, *p* > 0.05).

#### 3.5.2 Subgroup analysis of decision-making indicators

The results of subgroup analysis indicated that the reaction time of athletes aged 18–22 or over 22 did not show significant improvement (SMD = −1.68, *p* > 0.05; SMD = 0.1, *p* > 0.05). In terms of sports experience, athletes with ≥7.5 years showed a positive improvement in reaction time, which was statistically significant (SMD = −3.9, *p* < 0.001), while athletes with <7.5 years of sports experience did not show a significant improvement in reaction time (SMD = −0.39, *p* > 0.05). In terms of sports types, neither athletes in open sports events nor those in closed sports events showed significant importance in decision-making ability (SMD = 0.32, *p* > 0.05; SMD = −1.68, *p* > 0.05). In the stroboscopic frequency, <10 Hz and ≥10 Hz had no significant effect on decision-making ability (SMD = 0.1, *p* > 0.05; SMD = −1.68, *p* > 0.05). When the total intervention duration was ≤10 min, the decision-making ability of the athletes was significantly improved, which was statistically significant (SMD = −3.9, *p* < 0.001), while when it exceeded 100 min, the decision-making ability did not improve significantly (SMD = 0.19, *p* > 0.05). The intervention weeks of <4 weeks and ≥4 weeks did not improve the decision-making ability of the athletes (SMD = 0, *p* > 0.05; SMD = −0.94, *p* > 0.05). Training ≤2 times and ≥3 times per week did not improve the decision-making ability of athletes (SMD = −1.9, *p* > 0.05; SMD = 0.39, *p* > 0.05). Neither training sessions lasting less than 10 min nor more than 24 min can enhance the decision-making ability of athletes (SMD = 0, *p* > 0.05; SMD = −0.94, *p* > 0.05).

### 3.6 Heterogeneity and sensitivity analysis

The results of heterogeneity analysis in this study were as follows (Table 3). Strobe vision training showed a high degree of heterogeneity in the indicators of reaction time (I^2^ = 80%, *p* < 0.1) and a moderate degree of heterogeneity in the indicators of decision-making ability (I^2^ = 66%, *p* < 0.1). Due to the high heterogeneity, we speculate that the differences in outcome indicators might be the main source of heterogeneity. Therefore, a sensitivity analysis was conducted in the study to ensure the stability of the research results (Table 4).

**Table 3:** Subgroup analysis of reaction time (ms) performance indicators.

Outcomes			Studies (sample size)	Meta-analysis results			
				SMD	95% CI	Z	*p*
Reaction time	Participant age	≤18 Years	3 (122)	−0.32	(−1.34, −0.01)	1.99	0.05
		> 18 Years	4 (143)	−1.38	(−2.12, 0.06)	1.85	0.07
	Sport experience	< 7 Years	2 (100)	−0.35	(−0.74, 0.05)	1.72	0.09
		≥7 Years	2 (43)	−2.02	(−5.65, 1.62)	1.09	0.28
		Unclear	3 (122)	−0.68	(−1.34, −0.01)	1.99	0.05
	Sports type	Open-Skill	3 (130)	−0.60	(−1.15, −0.05)	2.15	0.03
		Closed-Skill	2 (43)	−2.02	(−5.65, 1.62)	1.09	0.28
		Unclear	2 (92)	−0.37	(−0.87, 0.13)	1.44	0.15
	Stroboscopic frequency	< 10 Hz	4 (148)	−1.38	(−2.54, −0.21)	2.32	0.02
		≥ 10 Hz	2 (100)	−0.35	(−0.74, 0.05)	1.72	0.09
		Unclear	1 (17)	−0.20	(−1.15, 0.76)	0.41	0.68
	Duty cycle	≤ 50%	4 (148)	−1.38	(−2.54, −0.21)	2.32	0.02
		> 50%	2 (100)	−0.35	(−0.74, 0.05)	1.72	0.09
		Unclear	1 (17)	−0.20	(−1.15, 0.76)	0.41	0.68
	Total intervention duration	≤ 10Min	3 (122)	−0.68	(−1.34, −0.01)	1.99	0.05
		> 100Min	4 (143)	−1.03	(−2.12, 0.06)	1.85	0.07
	Weeks of intervention	< 6 Weeks	3 (122)	−0.68	(−1.34, −0.01)	1.99	0.05
		≥ 6 Weeks	4 (143)	−1.03	(−2.12, 0.06)	1.85	0.07
	Intervention frequency	≥ 3Sessions	3 (117)	−0.32	(−0.69, 0.04)	1.74	0.08
		≤ 2Sessions	1 (26)	−3.90	(−5.29, −2.52)	5.52	<0.001
		Single Intervention	3 (122)	−0.68	(−1.34, −0.01)	1.99	0.05
	Per session intervention duration	< 10Min	3 (122)	−0.68	(−1.34, −0.01)	1.99	0.05
		≥ 25Min	4 (143)	−1.03	(−2.12, 0.06)	1.85	0.07

**Table 4:** Subgroup analysis of decision-making (% or s) performance indicators.

Outcomes			Studies (sample size)	Meta-analysis results			
				SMD	95% CI	Z	*p*
Decision-making	Participant age	18-22 Year	2 (56)	−1.68	(−5.96, 2.60)	0.77	0.44
		>22 Year	2 (90)	0.10	(−0.31, 0.51)	0.47	0.64
	Sport experience	<7.5 Year	2 (58)	0.39	(−0.13, 0.92)	1.49	0.14
		≥7.5 Year	1 (26)	−3.90	(−5.29, −2.52)	5.52	<0.0001
		Unclear	1 (62)	0.00	(−0.50, 0.50)	0.00	1.00
	Sports type	Open-Skill	1 (28)	0.32	(−0.42, 1.07)	0.85	0.39
		Closed-Skill	2 (56)	−1.68	(−5.96, 2.60)	0.77	0.44
		Unclear	1 (62)	0.00	(−0.50, 0.50)	0.00	1.00
	Stroboscopic frequency	≥4 Hz	2 (90)	0.10	(−0.31, 0.51)	0.47	0.64
		<4 Hz	2 (56)	−1.68	(−5.96, 2.60)	0.77	0.44
	Total intervention duration	>100 min	3 (120)	0.19	(−0.17, 0.55)	1.03	0.30
		≤10 min	1 (26)	−3.90	(−5.29, −2.52)	2.52	<0.0001
	Weeks of intervention	≥4 weeks	3 (84)	−0.94	(−3.03, 1.14)	0.89	0.38
		<4 weeks	1 (62)	0.00	(−0.50, 0.50)	0.00	1.00
	Intervention frequency	≥3sessions	2 (58)	0.39	(−0.13, 0.92)	1.49	0.14
		≤2sessions	2 (88)	−1.90	(−5.72, 1.93)	0.97	0.33
	Per session intervention duration	≥24 min	3 (84)	−0.94	(−3.03, 1.14)	0.89	0.38
		<10 min	1 (62)	0.00	(−0.50, 0.50)	0.00	1.00

**Figure 3:**
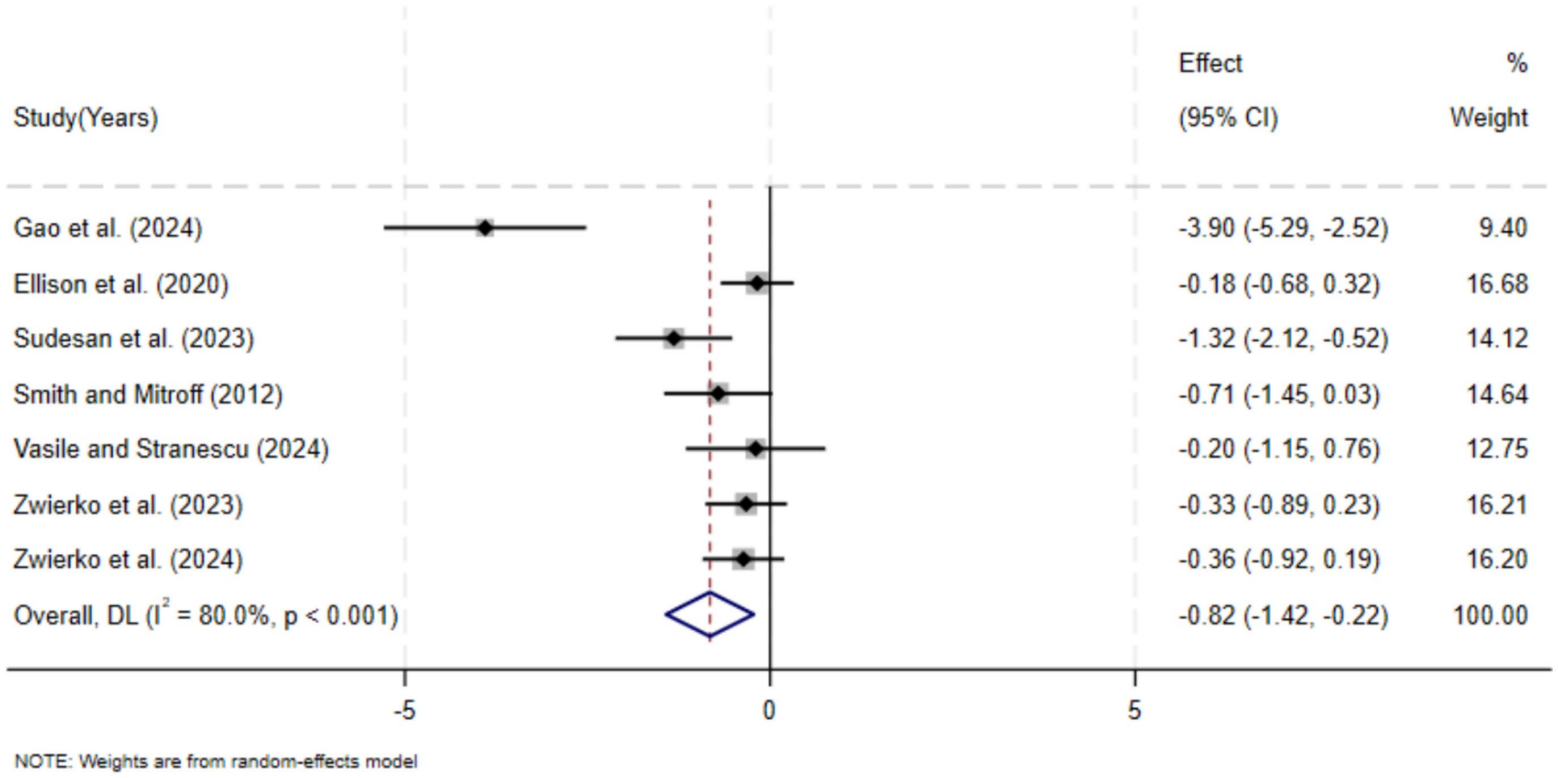
Meta-analysis forest plot of the effect of stroboscopic vision training on reaction time.

The sensitivity analysis of the study was tested by using the method of elimination one by one. The results indicated that, after excluding any individual studies, the point estimates of the combined effect size showed no substantial differences from the original results, and the confidence intervals still maintained statistical significance. This indicates that the main conclusion of this study that stroboscopic visual training can significantly shorten reaction time is highly robust (as shown in Figure 3).

**Figure 4:**
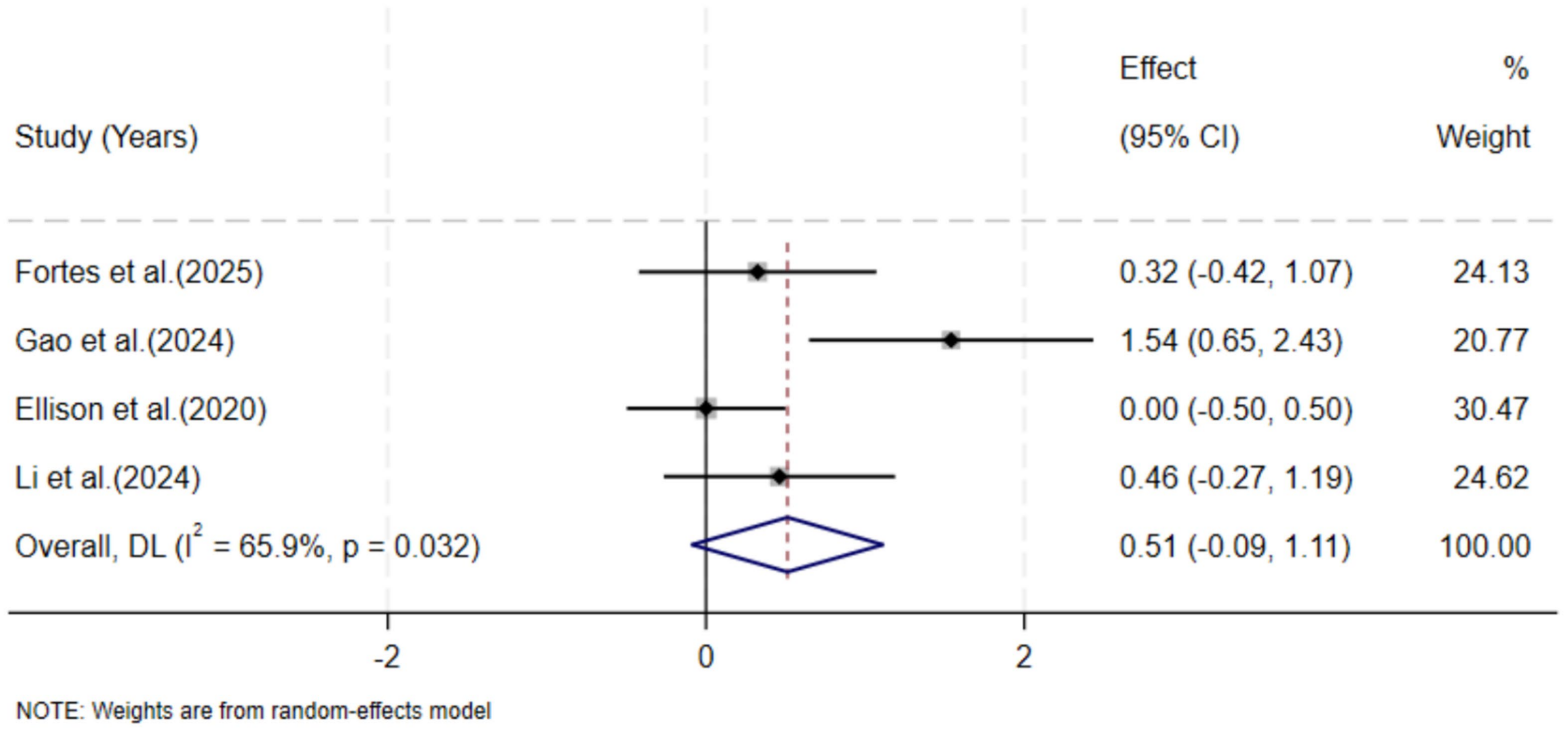
Meta-analysis forest plot of the impact of stroboscopic vision training on decision-making ability.

**Figure 5:**
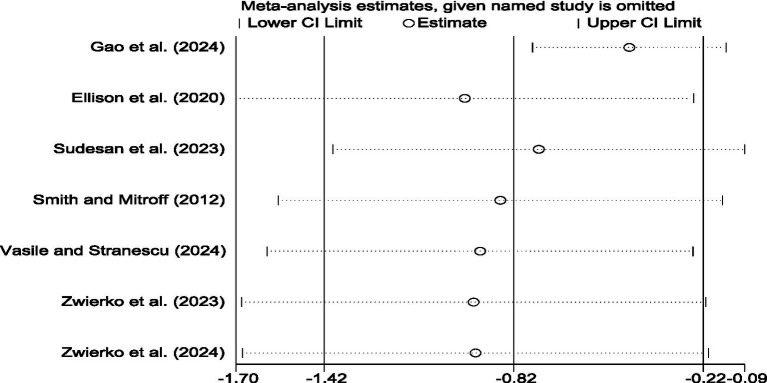
The results of the sensitivity analysis included in the research on reaction time indicators.

**Figure 6:**
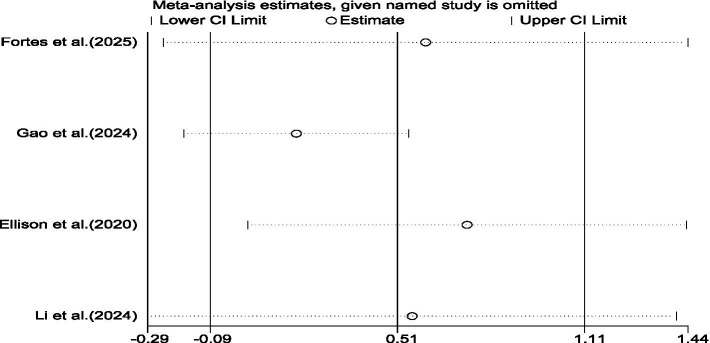
Shows the results of the sensitivity analysis included in the research on decision-making ability indicators.

The sensitivity analysis of the study was tested by using the elimination method one by one. The results showed that the combined effect size did not change directionally, and the point estimate fluctuated between 0.29 and 0.60, verifying that the trend of the positive effect was stable. However, after excluding Li et al. (2024), the effect size decreased, indicating that this study provides greater support for the overall effect (as shown in Figure 4; Table 5). Overall, stroboscopic training has shown an insignificant positive trend in decision-making ability, but the possible causes of this need further analysis (Figures 5, 6).

**Table 5:** Results of heterogeneity analysis.

Outcomes	Q	I^2^ (%)	*p*	SMD	95% CI	Z	*p*
Reaction	29.95	80%	<0.0001	−0.82	(−1.42, −0.22)	2.69	0.007
Decision-making	8.79	66%	0.03	0.51	(−0.09, 1.11)	1.67	0.09

### 3.7 Publication bias test

**Figure 7:**
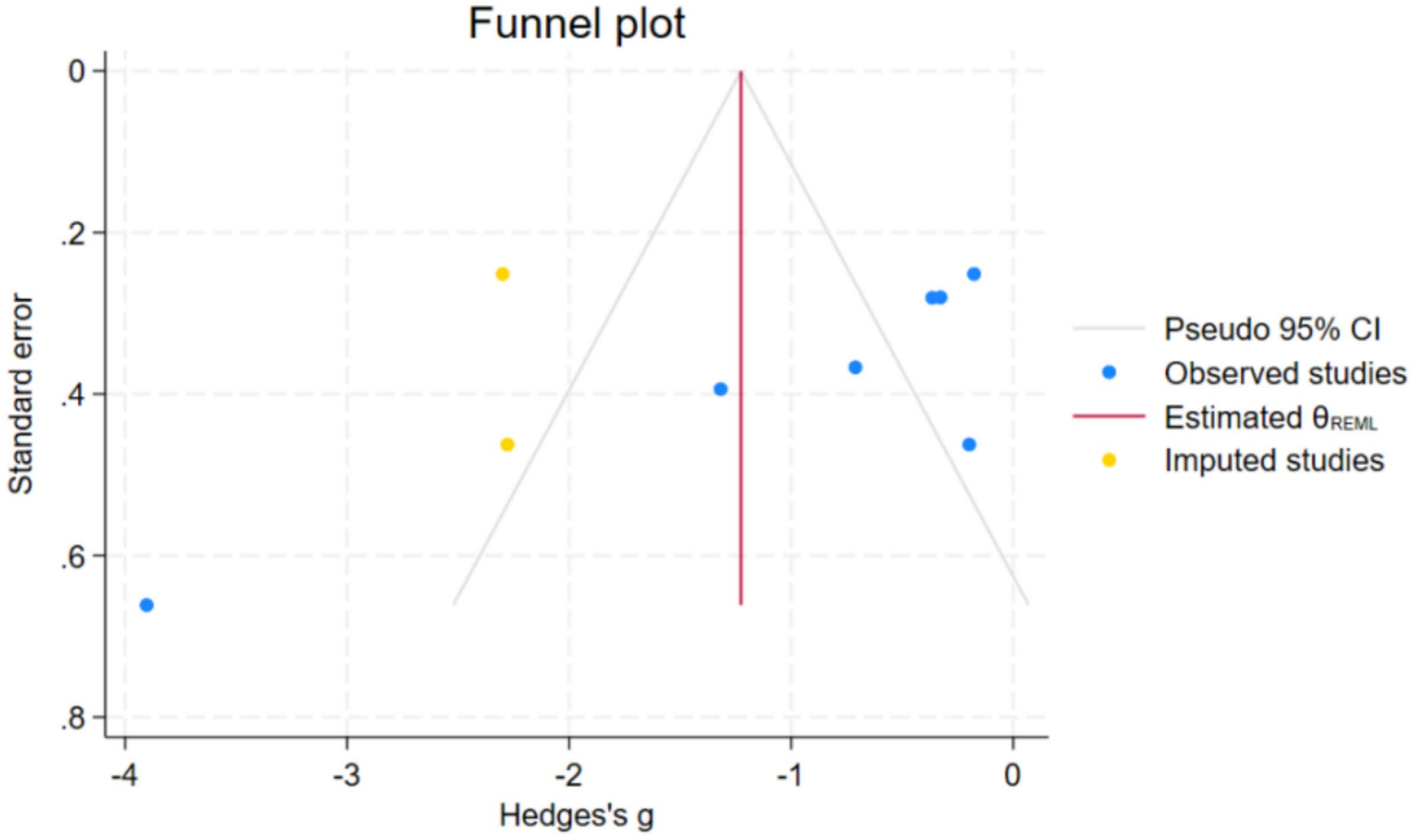
Publication bias in response time.

The study conducted a preliminary assessment of the publication bias of the response time index through funnel plots. Additionally, we performed Egger’s linear regression test, and the results showed that Z = −3.87, *p* < 0.05. This indicates the existence of statistical publication bias. Randomly, we use the subtraction and supplementation method for correction. The model estimated two potentially missing studies, with pre-adjusted effect values of Hedges’ g: −0.913, 95% CI: −1.778, −0.048. The adjusted effect values were Hedges’ g: −1.226, 95% CI: −2.011, −0.441. The significance and direction of the effect size have not changed, which supports the robustness of the results of this study (Figure 7).

**Figure 8:**
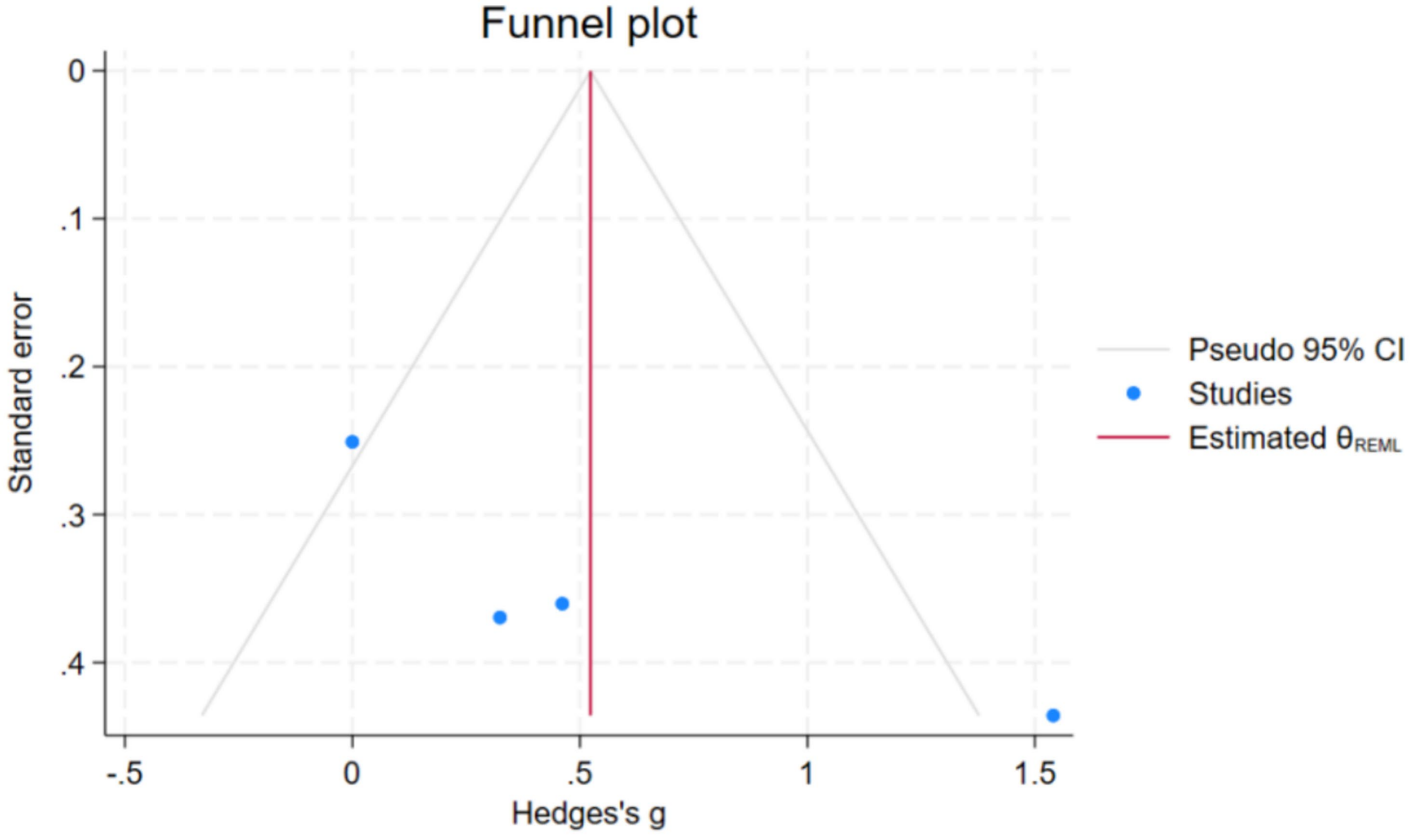
Publication bias in decision-making ability.

The study conducted a preliminary assessment of the publication bias of the indicators through funnel plots. In addition, we conducted Egger’s linear regression test, and the results showed that Z = 2.60, *p* < 0.05. This indicates the existence of statistical publication bias. Randomly, we use the subtraction and supplementation method for correction. The model estimated one potentially missing study. The pre-adjusted effect size was Hedges’ g: 0.523, 95% CI: −0.095, 1.141. The adjusted effect values were Hedges’ g: 0.746, 95% CI: 0.117, 1.376. The results indicated that publication bias might have led the original analysis to underestimate the true effect of the intervention and mask its significance (Figure 8).

## 4 Discussion

This study comprehensively evaluated the impact of stroboscopic vision training on athletes’ reaction ability and decision-making ability through a systematic review and meta-analysis. The main analysis indicates that stroboscopic vision training is an effective training method for improving athletes’ reaction time. However, the effect of stroboscopic vision training on improving the decision-making ability of athletes does not show a significant level. It is important to note that both analyses indicated some degree of heterogeneity and that there may be a multifactorial moderating effect of training effects.

### 4.1 The influence of SVT on athletes’ reaction time

With regard to SVT significantly improving the reaction time of athletes, this result is generally consistent with the results of several previous research reviews. Researchers believe that SVT is an advanced perception-cognitive training tool. SVT can significantly improve athletes’ specific cognitive abilities (visual and visual-motor functions, reaction time, anticipative judgment, etc.), and its effects can be transferred to real competitions after stroboscopic training (Wilkins and Appelbaum, 2020; Jothi et al., 2025). Research suggests that one important reason why stroboscopic training can significantly improve reaction time might be due to the neurophysiological mechanism of SVT activation. Because stroboscopic visual training intermittently deprives visual input, it forces the brain to allocate attention resources more efficiently under the condition of incomplete visual information, optimizing the extraction and processing speed of key visual information. The particularity of stroboscopic training enables athletes to rely more on short-term memory and prediction to track targets and predict the movement trajectory of the ball (Harris et al., 2025), and to rely more on prediction strategies to complete specific movement tasks. When adjusted to normal visual conditions, the adaptive changes in visual information processing and motion control will produce a compensatory effect, enhancing the coupling of perception and action. Athletes will exhibit more efficient cognition under conditions of a clearer visual environment and richer visual information. This explanation has been verified in rock climbing research, which suggests that stroboscopic visual training has a significant effect on enhancing the spatial transformation ability and reaction ability of rock climbers (Vasile and Stănescu, 2024).

In addition, SVT significantly improved the working efficiency of the dorsal visual pathway (Tzagarakis et al., 2010). Studies have further revealed through brain waves that the interference of stroboscopic training shortens the latency of P100 in visual evoked potentials, enhances neuronal synchronization, and the improvement of neural efficiency directly leads to the improvement of reaction speed (Wilkins et al., 2018). Meanwhile, the beta band connectivity in the sensorimotor circuit is enhanced, which is closely related to the multi-sensory reweighting process and directly affects visual-motor perception, enabling athletes to exhibit more stable visual performance during competition (Cooke and Bliss, 2006). Furthermore, when researchers analyzed the impact of stroboscopic training on prediction time, they found that the reaction speed of athletes after SVT was better than that of the control group (Smith and Mitroff, 2012). In another study on football goalkeepers, their visual reaction time improved significantly after 7 weeks of stroboscopic training. However, no good training effects were found in terms of attention and hand-eye coordination (Bennett et al., 2018). Overall, our research supports the existing consensus, and the arguments of previous studies provide strong evidence for our systematic review and meta-analysis.

First of all, different stroboscopic vision training programs have different effects on athletes’ reaction time, and sometimes even opposite results. Therefore, we must pay attention to the differentiated impact of the frequency of stroboscopic glasses and the duration of stroboscopic vision training on cognitive ability. This study found that the setting of stroboscopic parameters with low frequency and low duty cycle can significantly improve the response capability. This supports the core of the research: SVT induces neural adaptation by increasing task difficulty, because lower frequencies and duty cycles mean longer visual interruption times and greater perceptual challenges (Schroeder et al., 2011; Clark, 2020). On the contrary, excessively high stroboscopic frequencies or duty cycles are very similar to normal visual conditions, and visual interference may fail to cause athletes to undergo perception-movement adaptive changes. Therefore, appropriate visual interference is crucial for the improvement of cognitive ability. When conducting stroboscopic training, we recommend starting with the shading settings for humidity. If the task is difficult, the duty cycle can be increased or the frequency raised to reduce the training difficulty. Conversely, for less difficult training, the duty cycle can be lowered or the frequency increased to enhance visual stimulation and thereby improve the training effect. It is also worth noting that both single short training sessions and 1–2 training sessions per week have improved reaction ability. However, the intrinsic mechanisms by which the two training programs improve athletes’ reaction abilities may be completely different. A single short training session may trigger an acute or preheating effect in the nervous system, while 1–2 training sessions per week represent true long-term training adaptation. This indicates that the duration of stroboscopic training is not the longer the better, nor is it the more frequent the better. Studies show that prolonged training may not effectively improve reaction ability, and overly frequent training may also reduce positive effects due to fatigue accumulation (Luo et al., 2025). Overall, stroboscopic training for 1 to 6 weeks, with 1 to 2 sessions per week and 10 min of high-quality training each time, is an excellent training plan for optimizing an athlete’s reaction ability.

Secondly, regarding the differences among the participants, the research found that stroboscopic training improved the reaction ability of adolescent athletes to a greater extent than that of adult athletes. This might be due to the fact that adolescent athletes are in a crucial period of neuro-development, which may induce deeper and longer-lasting adaptive changes. It leads to neuroplastic changes in brain function (Nozari et al., 2020; Lochhead et al., 2024; Hülsdünker et al., 2021a). The study did not find a direct relationship between training experience and reaction ability. Regardless of whether the training experience was more than 7 years or less than 7 years, the reaction time of athletes did not improve significantly. This point is at odds with previous studies. However, studies have shown that for novice athletes, stroboscopic vision training may improve their perception-decision-making skills, while for experienced athletes, stroboscopic training is mainly for maintaining and improving skills (Davids et al., 2003; Tobin, 2021). We speculate that the reason for the ambiguity might be that previous studies did not screen athletes based on the definitions of “experts” and “novices.” Therefore, researchers must take a crucial step in defining experts and novices and how to precisely select participants in the future.

Finally, in different types of sports, the improvement effect of the reaction ability of open-skill athletes (football, volleyball) is better than that of closed-skill athletes. Long-term specific training for open-skill athletes can increase the number of Purkinye neurons and synapses, promote angiogenesis in the prefrontal cortex, activate the sensorimotor network related to cognitive ability, and improve neural function (Hu et al., 2025). This might be because open-skill athletes usually need to train in a constantly changing environment, which requires them to remain highly vigilant. The unpredictability and complexity of this environment force the brain to constantly update information, which on the one hand improves reaction time and on the other hand enhances working memory capacity. In conclusion, stroboscopic vision training has indeed made significant improvements to athletes’ reaction abilities, but it also has some limitations. We believe that stroboscopic vision training can be used as a commonly employed tool to enhance athletes’ reaction capabilities.

### 4.2 The influence of SVT on athletes’ decision-making ability

However, the research did not find a significant positive impact of stroboscopic vision training on athletes’ decision-making ability. Decision-making is a more advanced and complex cognitive function than simple reactions. It involves environmental prediction, weighing multiple options, risk assessment, etc. The improvement in basic visual processing speed brought about by stroboscopic vision training may not be directly and linearly translated into the enhancement of decision-making ability and advanced cognitive ability (Fransen, 2024). Some studies have examined the impact of stroboscopic vision training on subsequent perception (near transfer) and sport task performance (far transfer). A large amount of research evidence indicates that stroboscopic vision training can lead to near transfer of perception-cognitive functions (such as reaction agility, short-term memory, expected reaction time, etc.), but the evidence for far transfer of sport performance remains uncertain (Harris et al., 2018; Beavan et al., 2021). For instance, we can expect that football juggling skills training might make an athlete a very good juggler, but it may not have any impact on the athlete’s balance ability. Current studies have pointed out that stroboscopic vision training does not support the generation of distant migration in ice hockey, badminton and football (Mitroff et al., 2013; Hülsdünker et al., 2019; Palmer et al., 2022). Furthermore, some researchers have proposed that the effect of stroboscopic vision training in optimizing sport performance is influenced by significant differences in research designs (Carroll et al., 2021). Overall, although stroboscopic glasses increase the difficulty of visual training, there is no conclusive evidence that training under intermittent visual occlusion conditions promotes the distant transfer of sport performance (Fransen, 2024).

Research has found that different training experiences have an impact on decision-making. Sports experience plays a crucial role in the decision-making of specific sports. Experienced athletes, due to their rich professional knowledge reserves, can demonstrate superior perception-cognitive abilities and decision-making performance. Excellent anticipation ability can enable athletes to alleviate the time constraints of tasks, perceive and process competition-related information earlier, and make faster and more accurate decisions. In addition, research shows that a shorter intervention period has a positive effect on decision-making (Fransen, 2024). This is similar to the reasons for the effect on reaction ability. We suggest that in practical applications, the stroboscopic training plan needs to be adjusted at any time according to the specific combat readiness goals. For instance, in the preparation for the new season, appropriately increasing the number of weeks of SVT can enhance accuracy. In the pre-competition preparation, appropriately increasing the training frequency can consolidate the reaction ability. SVT can promote the improvement of perception-decision-making ability for young athletes, while for outstanding adult athletes, its main function is to maintain reaction and decision-making ability.

## 5 Limitations and future research directions

Although the research results support the effectiveness of stroboscopic training to a certain extent, there are still certain limitations. Firstly, the number of included studies was relatively small, the research results on SVT are still preliminary and need to be repeatedly verified using larger samples in different sports fields. Secondly, the limited number of studies for subgroup analysis increases the risk of effect size analysis. Although an analysis of the sources of heterogeneity was conducted, there may still be other factors influencing the research results. Finally, does stroboscopic training demonstrate distant transfer, that is, can stroboscopic training enhance athletic performance and how can it be transformed from an improvement in cognitive ability into athletic performance in real competitions? Future research needs to further discuss how to efficiently utilize stroboscopic training. Firstly, athletes of different sports levels or experiences may need personalized stroboscopic training tailored to their specific needs. Additionally, the requirements for stroboscopic training in different sports or types of events also need to be taken into account. Secondly, due to the limitations of the research language and search, relevant studies may be overlooked. In the future, it is necessary to optimize the search strategy and technology application, collect as comprehensive a dataset as possible, and ensure the robustness of the research. Finally, it is necessary to further analyze the applicability of stroboscopic training and its potential internal mechanisms, and strive to provide assistance for practical applications.

## 6 Conclusion

This meta-analysis demonstrates that stroboscopic training significantly enhances athletes’ reaction ability. Firstly, an optimal training protocol involves 1–6 weeks of intervention, with 1–2 sessions per week and approximately 10 min per session. Secondly, Lower frequency and duty cycle settings are more effective, while excessive visual disruption may diminish benefits. Thirdly, SVT has a significant enhancing effect on the reaction ability of open-skill sports, but no improvement in the reaction ability of closed-skill sports was found. In addition, SVT did not significantly improve the decision-making accuracy for either skill sports, which we believe is most likely related to the scarcity of the number of articles that included the accuracy. Fourth, Participants under 18 years also showed notable improvement. Finally, stroboscopic training had minimal effect on decision-making ability, except when applied briefly among experienced athletes. These findings offer practical guidance for implementing stroboscopic training. Future research should focus on elucidating its neural mechanisms and further validating its transfer effects to sport-specific decision-making performance.

## Data Availability

The original contributions presented in the study are included in the article/supplementary material, further inquiries can be directed to the corresponding author.

## Appendix 1

Search strategy.

**Table:**

Data	Query	Results
Pubmed	((((((Stroboscopic Training) OR (Stroboscope visual training)) OR (Stroboscope training)) OR (Strobe training)) )) AND ((((((((Perceptual-cognitive) OR (Visual function)) OR (Visuomotor reaction)) OR (Reaction speed)) OR (Reaction agility)) OR (Decision-making)) OR (Sports performance)) OR (Athletic performance))) AND ((Athletes) OR (Professional athletes))	52
Web of Science	TS =(Stroboscopic Training OR Stroboscope visual training OR Stroboscope training OR Strobe training) AND TS=(Perceptual-cognitive OR Visual function OR Visuomotor reaction OR Reaction speed OR Reaction agility OR Decision-making OR Sports performance OR Athletic performance) AND TS =(Athletes OR Professional athletes)	28
Embase	(('stroboscope'/exp OR stroboscope) AND visual AND ('training'/exp OR training) OR (('stroboscope'/exp OR stroboscope) AND ('training'/exp OR training)) OR (strobe AND ('training'/exp OR training))) AND ('perceptual cognitive' OR (visual AND ('function'/exp OR function)) OR (('motor'/exp OR motor) AND cognitive) OR (('sport'/exp OR sport) AND ('vision'/exp OR vision)) OR (visuomotor AND ('reaction'/exp OR reaction)) OR (('reaction'/exp OR reaction) AND ('speed'/exp OR speed)) OR (('reaction'/exp OR reaction) AND ('agility'/exp OR agility)) OR 'anticipation'/exp OR anticipation OR 'attention'/exp OR attention OR (visual AND ('memory'/exp OR memory)))	68
Scopus	TITLE-ABS-KEY (Stroboscope visual training) OR TITLE-ABS-KEY (Stroboscope training) OR TITLE-ABS-KEY (strobe training) AND TITLE-ABS-KEY (perceptual-cognitive) OR TITLE-ABS-KEY (visual function) OR TITLE-ABS-KEY (motor cognitive) OR TITLE-ABS-KEY (sport vision) OR TITLE-ABS-KEY (visuomotor reaction) OR TITLE-ABS-KEY (reaction speed) OR TITLE-ABS-KEY (reaction agility) OR TITLE-ABS-KEY (anticipation) OR TITLE-ABS-KEY (attention) OR TITLE-ABS-KEY (visual memory)	61
EBSCO	(Stroboscopic Training OR Stroboscope visual training OR Stroboscope training OR Strobe training) AND (Perceptual-cognitive OR Visual function OR Visuomotor reaction OR Reaction speed OR Reaction agility OR Decision-making OR Sports performance OR Athletic performance) AND (Athletes OR Professional athletes)	18
